# Refugees’ Encounters With Nordic Rural Areas–Darkness, Wind and “Hygge”!

**DOI:** 10.3389/fsoc.2021.623686

**Published:** 2021-03-15

**Authors:** Lise Herslund, Gry Paulgaard

**Affiliations:** ^1^ Department of Geosciences and Natural Resource Management, University of Copenhagen, Copenhagen, Denmark; ^2^ UiT The Arctic University of Norway, Tromsø, Norway

**Keywords:** refugees, rural, nordic, everyday lives, weather, commuting, local volunteers

## Abstract

The paper investigates how refugees settled in rural Norway and Denmark experience and interact with their new rural places of residence. Theoretically, the paper finds inspiration in “phenomenology of practices” (Simonsen, Prog. Hum. Geogr., 2012, 37, 10–26), which emphasizes the bodily and sensory experiences of daily life that spur feelings of, for example, “orientation” or “disorientation”. The empirical material is based on fieldwork and qualitative interviews with refugees and local volunteers in 2016/2017/2019 in small towns in the rural north of Norway and rural Denmark. There are several differences between the Norwegian and Danish rural areas, in relation to distances, climate and population density. Nonetheless, the ways in which the rural areas are experienced from within, by refugees settled there, show surprisingly many similarities. Many of the informants, in both the Norwegian and Danish cases, initially expressed frustration at being placed in rural areas without having any say in the matter. Those who were former city-dwellers especially experienced moments of disorientation, as their encounters with Nordic rural life were experienced as the opposite of their urban backgrounds. Limiting structural conditions very much shape the everyday lives of refugees in the first years, when they do not have a car or the financial capacity to find their own house. They feel stressed, with busy everyday lives made up of long commuting hours on public transport. In these first years of uncertainty, the dark and harsh weather very much adds to the feeling of stress and insecurity. What seem to add “orientation” are social relations with other refugees and local volunteers organizing activities.

## Introduction

The number of international migrants is increasing in rural areas. This is particularly the case in the Nordic countries, where the rural population is becoming more diverse than the EU average (Nørregaard, 2018). Here, dispersal policies have led refugees to regional towns and rural areas over recent decades (Larsen, 2011; Søholt et al., 2018). However, a large number of the refugees initially placed in rural areas in both Denmark and Norway have moved toward city areas over the years and also to a larger degree than other international migrants (Andersen, 2015; Ordemann, 2017). This paper explores the experiences of refugees arriving in rural areas in Denmark and Norway during the large influx of refugees in 2015/2016.

There is a debate as to whether refugees should be settled in rural areas at all. An argument for settling international migrants in rural areas has been that migrants can act as an important means for rural re-population, and many peripheral municipalities actively try to attract international migrants to their communities (Hedberg and Haandrikman, 2014; Aure et al., 2018; Nørregaard, 2018; Søholt et al., 2018; Woods, 2018). However, several authors dispute the idea that refugees could be catalysts for peripheral development, when rural areas lack jobs as well as the resources and services to adequately cater for their needs (Wren, 2003; McAreavey and Argent, 2018; Woods, 2018). Urban areas are often portrayed as heterogeneous, providing an opportunity to “blend in” and be anonymous (Massey, 2007). Rural areas, on the other hand, have been described as places where being a refugee and migrant can be difficult, due to the pressure to conform to the culture and norms of the dominant majority (de Lima, 2012; Kelly, 2013; Eriksson et al., 2015; Rysst, 2017; ). Rural areas have often been portrayed as homogenous, safe and stable, on the one hand, and boring, backwards, with a high degree of social control, on the other (Rye, 2006; Paulgaard, 2008). The view of rural areas as homogenous, cohesive and good places to live and grow up is deeply seated in the Norwegian and Danish social imaginaries (Gullestad, 2002; Mathisen, 2020). The belief that rural areas represent good places to live and that their stronger associational life could make it easier for refugees to integrate has been one of the reasons for adopting dispersal policies and settling refugees in rural areas (Larsen, 2011). Thus, rural social life whether promoting social control or social integration is another key factor in the debate on whether refugees should be placed in rural areas.

This paper aims to contribute to the debate by exploring how refugees settled in Nordic rural areas experience and interact with their new rural places of residence in their daily lives. The empirical material is based on fieldwork and qualitative interviews with refugees and local volunteers in 2016/2017/2019 in small towns in rural Norway and Denmark.

The paper finds that refugees in rural areas struggle with limiting structural conditions like long distances, limited employment possibilities and lack of affordable rental housing. Social relations with other refugees, immigrants and the local communities can help counter some of the problems of living in an unfamiliar environment and a rural setting. However, besides the social life and structural conditions, the more “physical” aspects of rural life-the material surroundings, including circumstances like the weather-affect refugees’ experiences. Although there are great differences between the climate in northern Norway and Denmark, the refugees arriving in both areas were all quite affected by the harsher weather. This condition seems to be somewhat overlooked or found unimportant in other studies. The experiencing of nature, going out in all kinds of weather, taking walks and getting fresh air also play an important part in the “good life” in the Nordic countries. In Norway, such cultural beliefs regarding the importance of outdoor life are even evident in the school curriculum (Ødegaard and Marandon, 2019). Thus, in this study, we also bring forward the way in which the physical surroundings and the weather are experienced by the refugees.

Theoretically, we find inspiration in “phenomenology of practices” (e.g., Simonsen, 2012), which stresses the insufficiency of describing the world’s structures without also paying attention to the way they are experienced from within.

## Theoretical Approach

### Refugees’ Phenomenology of Practice

In this paper, we take a starting point in the phenomenology of practice, which situates practical, embodied consciousness in the world: an “inter world”, where meaning and materiality are inseparable (Simonsen, 2012:15). Materiality refers first and foremost to the examination of people’s contact with their physical surroundings, taking into account that agency is constructed through material engagement in social practices. The materiality of a place is a part of the embodied nature of being (Lähdesmäki et al., 2016). Such a perspective implies acknowledging the interdependency between the social and material contexts for practice. The emphasis on materiality, on “the non-human and more than human” (Simonsen, 2012:21), gives space to nature and objects, without reducing one to the other. The encounters with physical objects, as well as surroundings, social relations and structures, constitute sensory experiences that include the situated body and the body as lived experience (Lødding and Paulgaard, 2019).

Simonsen (2012) focuses on how the world’s structures are experienced from within. She describes how “active bodies” use their acquired schemes and habits to position their world around themselves. Such bodies are dynamic in measuring space in the construction of a meaningful world:

Inhabiting space is both about “finding our way” and how we come to “feel at home”. It therefore involves continuous negotiation between what is familiar and what is unfamiliar, making space habitable but also receiving new impressions depending on which way we turn and what is in reach. (Simonsen, 2012:16)

Simonsen (2012) also stresses that “bodies” are different and describes how immigrants can be blocked and stopped in their everyday life because of how they look: their “visibility”. She describes a process of “othering” when immigrants are, for example, stopped when entering a nightclub, making them feel different and out of place. How such embodied difference shapes the meeting and engagement with their new places and how meanings form around these differences is a central concern.

(Kinkaid, 2020:169) points to the fact that “Difference is not located in space itself or in essential characteristics of bodies or things; rather, “differences” are formed through lived practice; sedimentation of experience”. In order to understand the production and embodiment of difference, it is important to study the way embodied sedimentations form and delimit “the subject of difference”. Such an approach will illuminate how different kinds of bodies encounter space differently. Kincaid uses the term “contradictions of space”, referring to a moment occurring within the experience of a subject, when he or she does not, or cannot, practice space properly. In situations where the relation between the subject and the milieu fails to cohere, then the general background of perception and understanding, the acquired and embodied knowledge and competence, can be called into question: “Space becomes contradictory rather than synthetic, the body becomes alienated, an object in space” (2020:180). Being a refugee implies that acquired schemes and habits are not always useful in new contexts, and it is through everyday encounters that this is experienced. Such experiences can result in both spatial and social disorientation, “As the normative meanings and practices they know from home cannot be used” (Kinkaid, 2020:180).

According to Simonsen (2012), orientation and familiarity are connected to situations where the phenomenological body gains the capacity to orient itself in one way or another. Orientation is about both “finding our way” and “feeling at home”. Familiarity is neither delimited nor static; Simonsen points out the dynamic aspect of familiarity continuously in formation. This relates to the understanding of the phenomenological body as dynamic and always in process, continuously weaving meaning throughout the course of its existence, in interaction with others and with its environment. Such a phenomenology of practice situates practical, embodied consciousness in the world: an “interworld”, where meaning and materiality are inseparable (Mathisen, 2020; Simonsen, 2012:15).

Based on Ahmed (2006), Simonsen points out that orientation also involves moments of disorientation, similar to Kinkaid, (2020) “contradiction of space”. Moments of disorientation might turn our world upside down. Such a feeling can shape insecurity and shatter one’s sense of confidence in the foundation of one’s existence. In such situations, support is needed to reground or re-orientate the relation to the world. According to Simonsen, (2012), moments of disorientation can be seen as destabilizing and undermining, but they can also be seen as productive moments, leading to new hopes and new directions. Spatial practice-the way our bodies move through the world using acquired schemes and habits-can, depending on how spaces are inhabited (when talking about immigrants, spaces that they were not intended to inhabit) and how the people that perform the practice meet, either reinforce dominant meanings and bodies or possibly open the door for new practices and spaces to emerge (Kinkaid, 2020).

### Encountering the “Physical” World-Climate and Weather Conditions

The phenomenology of practice situates practical, embodied consciousness in the world, where meaning and materiality are inseparable (Simonsen, 2012:15). Materiality refers to the physical surroundings, but does it also include the weather? For inspiration on how to understand materiality and physical surroundings and their role in the phenomenology of practice, we look toward Ingold, (2010), who criticizes theories and thinking about the material world as comprising the two broad components of landscape and artifacts. He claims that much attention has been paid to the ways in which people engage with the things of this world, to the apparent capacity of things to act back, and to the “hybrid” agencies that are formed when persons and things combine in the production of effects. One example is the centrality of the weather to life and experiences; nevertheless, in the scholarly literature, scarcely a word is to be found on the question of how the weather impacts on our daily life practices and experiences:

Much has been written on the perception of landscape; virtually nothing on the perception of the weather. It is extraordinary that something that has such massive impact on people’s activities, moods and motivations, indeed on the whole tenor of social life, has been so little considered. (Ingold, 2005:100)

Ingold claims that the failure to recognize the importance of the weather, not in human daily lives but in social theories, has to do with the lack of any conceptual framework within which to accommodate anything as protean and temperamental as the weather. He relates this to the fact that most scholars have considered materiality to be locked up in the congealed forms of the landscape and the solid objects resting on its surface-“on the hard physicality of the world” (Ingold, 2010:132). Ingold points out that such a conclusion is absurd. To draw the limits of materiality around the surfaces of the landscape and artifacts would be to leave the inhabitants of the landscape and artifacts in a vacuum. Ingold refers to (Gibson, 1979:106) and describes the air as a medium, stating that the quality of interaction will be tempered by what is going on in the medium, that is, by the weather (Ingold, 2010:133). According to Ingold, if the weather conditions our interaction with people and things, then it also conditions how we know them.

Migration can be described as a process of both disorientation and reorientation, as bodies both “move away” and “arrive” (Simonsen, 2012), and different bodies might encounter space differently (Kinkaid, 2020). When arriving in the Nordic areas, how is space the new town of residence experienced? What experiences result in feelings of meaning and belonging, familiarity and orientation and making the space habitable, and what experiences spur feelings of disorientation and contradiction? In the following, we will focus on the primacy of encounters, “of bodily encounters in all their complexity both structural, social and physical” (Simonsen, 2012:12).

## Materials and Methods

This paper investigates the experiences of refugees settling in rural areas in Northern Norway and Denmark in 2015/2016. The empirical material is mainly based on fieldwork and qualitative interviews with refugees and local volunteers who have started up activities for refugees in the local areas.

### The Norwegian Case

The Norwegian case takes its cue from the situation that occurred in the autumn of 2015 in the north of Norway, a region often termed “the marginal edge of the northern periphery”. In the course of a few months, over 5,500 migrants from 35 nations–mostly from Syria (40%), Afghanistan, Iraq and Iran–crossed the Russian-Norwegian border into Eastern Finnmark, the northernmost county in Norway. Many of those who came through this Arctic migration route were settled in small rural places in the north of Norway (Integrerings og mangfoldsdirektoratet (IMDi), 2019.

Qualitative interviews started at the beginning of 2016 at a refugee camp near the Russian border. Ten families, both mothers and fathers applying for asylum status in Norway, were interviewed. After the first period in the refugee camp, the families were granted asylum and three of them were settled in the same area in the northern part of Norway. Through the contact with these families, the research team established contact with other refugee families in the same area. The selection of informants followed the snowball method. The core of the informants were families who came from Syria, and these informants led to other refugee families and informants. The Norwegian case consists of a study of three small places in the same area, one with around 1,000 inhabitants, another with 2000 inhabitants, and the third with around 5,000 inhabitants.

The research is based on fieldwork with eight families living in these three places in the north of Norway. The families had from eight to two children, and most of the interviews and field conversations were carried out with the mothers and the oldest children. The fieldwork period is from two to four years, as some of the families have been contacted since the beginning of 2016. This gives the possibility to follow the process of finding one’s place and settling down in a new country.

### The Danish Case

The Danish case also takes its starting point in the flows of refugees coming, especially from Syria, up through Europe in 2015, when all European countries saw a marked rise in the numbers of refugees arriving. In Denmark, more than eight times more refugees arrived in 2015 than in earlier years (UNHCR, 2020). Some of these refugees ended up being resettled in rural areas.

In the Danish case, a total of seven towns of different sizes across four rural municipalities spread out across Denmark were selected: two towns with around 4,000 inhabitants and towns of 1,500, 1,100, 900, 800 and 600 inhabitants, respectively. In the Danish case, the key criteria for the selection of towns were 1) where refugees granted asylum had been placed following the influx of refugees in 2015, and 2) the extent to which the local community had initiated activities for them.

Interviews were conducted with a number of refugees resettled in these towns, local volunteers initiating activities for refugees, and key formal stakeholders, for example integration officers and NGO (Non-Governmental Organisations) representatives. The selection of refugees also followed the snowball method.

In Denmark, a total of 19 interviews with refugees were conducted, 15 of them with a single family or a single person; the rest were group interviews with 5–10 people. Five of the interviews constitute re-interviews, a year after the first interviews with refugees, across five of the towns. All interviews focused on themes such as refugees’ everyday life, their use and view of the town, their social relations, their wishes, and any plans, for the future.

### Case Presentations

Often the Nordic countries are treated as one entity. In much of the literature, the “Nordic rural area” is a commonly used term. What is common across the Nordic countries is the welfare state, and in Nordic migration studies the integration of immigrants and refugees into the welfare state has been a central research concern (Emerek, 2003; Jöhncke, 2007). But the Nordic rural areas also have several differences, especially in climate, geography and spatial distances. In the case of Northern Norway, it is three times the size of the whole of Denmark.

Norway is the northernmost country in Europe. In 2019, the total number of inhabitants was 5,328,212, while the country’s total land area is around 325,000 square meters. The number of inhabitants in Denmark is 5,784,188, and the land area is about 43,084 square kilometres. Because of the size of the countries and the number of inhabitants, the density of the population is higher in Denmark than in Norway. Most of the migrants, as well as refugees, arriving in both Denmark and Norway live in urban areas, but both countries have refugees settled in rural areas (Larsen, 2011; Integrerings og mangfoldsdirektoratet (IMDi), 2019; Mathisen, 2020).

Norway has a strategy for settling refugees across the country. The settling is based on collaboration between the central government and the Association of Norwegian local Land and regional Authorities. The initiative comes from the central government, asking municipalities across the country to accept refugees for settlement, and the municipal councils can decide whether they have the capacity to settle a suggested number of refugees, according to economic and housing opportunities. Municipalities that settle refugees receive economic support for the first five years a refugee is settled. The municipalities must provide the first housing and an obligatory two-year introductory program for adults (Mathisen, 2020). The introductory program in Norway consists of language training and lessons about Norwegian society. The participants in the program are also given the opportunity to practice language in a workplace. The municipality is responsible for providing a working environment for the newcomer, in order to practice language. The place for language practice does not have to be a place where the person wants to work in the future. Toward the end of the program, the refugee is expected to participate in a work program, designed to increase the chances of getting a job or to continue education ().

Denmark has had a strategy to disperse refugees across the country since 1999. According to the Danish Ministry of Integration, the aim of the spatial dispersal policy is to secure a better geographical distribution of new refugees and promote their integration into Danish society, by reducing their risk of becoming socially and economically marginalized in urban ethnic ghettos (Larsen, 2011).

In Denmark, similarly to Norway, it is the municipality in which the refugees are settled that has the main responsibility for their integration. The municipality must cater for the refugees’ integration for a period of three years, by offering language classes and, later, job training. It is also the responsibility of the municipality to find housing (Larsen, 2011). In both Denmark and Norway, the refugees are permitted to leave the place where they have been settled before the introductory period has ended; however, the refugees might then lose their rights, such as the municipality having to provide suitable housing, etc.

### Analysis of Data

The data from the two case studies (Danish/Norwegian) were initially analyzed as two separate studies. In both countries, the interview data were analyzed first through the use of an open approach, searching for common themes and points across the interview transcripts and summaries. Then, more focused thematic analyses were performed, following themes such as refugees’ everyday life, their meeting with their new place of residence, their use and view of the new town, their social relations, their plans for the future, etc. The two country analyses were then initially compared through a discussion of main points found and how they played out in the Danish and Norwegian cases, respectively. This discussion led to a listing of key points to unpack and compare: a list that resembles the structure of the following “results” section.

## Results

### Feeling Powerless and Different From the Start

The placing and settling of refugees in rural communities are matters of political and multi-level governance. In both the Danish and Norwegian cases, the refugees had little or no influence over where they were settled, and most felt powerless, as they could not choose themselves but had to be placed without any say in the matter. It made them feel like second-class citizens. A young married Syrian woman in Denmark said: “*I have an uncle in Århus and have made friends in the refugee camp with other refugees now settled near Ålborg. We could help each other out, but now I have to be settled in this little town away from everything … I am so frustrated and feel like cattle*.”

In both the Danish and Norwegian cases, most refugees came from Syria (2/3), with the rest coming from Eritrea and Somalia, then Afghanistan and Iraq. The Syrian refugees were often city-dwellers and, in the interviews, particularly refugees from Syria stressed that they preferred to live in cities because that was where they came from and were used to. “*We are city people, and we only know how to live with life around us*,” the father of a Syrian family said. On the contrary, the Eritreans and Somalis were often from villages. The refugees who came from urban areas had considerably more formal education than the adults from rural areas, some of whom had no formal education; thus, the Eritreans and Somalis were educated to a lesser extent.

In both Norway and Denmark, the refugees with city backgrounds felt frustration when they found out they were to be placed in a small town. At the refugee camps in both Denmark and Norway, the stories among the refugees were that being placed in rural areas after obtaining asylum would be dark and lonely, and there would be no jobs. The refugees from rural areas were, however, more content with being placed in rural areas. Some of the mothers from rural areas in Syria and Somalia stated that they liked to live in small places and reported that they quickly got into a daily routine. They joined up with other refugee women, with whom they met several times a week, prepared food, talked and took care of the small children. Their older children, on the other hand, could hardly wait to finish secondary school so they could move to bigger places–similar to many of their local Norwegian and Danish peers living in the same place.

### Difficult Everyday Movement-Experiencing Distance

In both the Danish and Norwegian cases, it was particularly the very stretched-out everyday life with long commuting hours that refugees initially experienced as stressful, overwhelming and also confusing. The former city-dwellers felt it especially hard, as they were used to being close to everything. Their initial “schemes and habits” of moving around in a denser city were very different from their new everyday life, with long commutes on public transport. In the Danish case, the main municipal town could often be reached within half an hour, so here it was more that the public transport was infrequent. In the Norwegian case, distances were huge, and going to the main municipal town would mean traveling for several hours, and public transport was infrequent.

Even though the distances varied in the two countries and across areas, the language classes refugees initially had to attend were, in all cases, centralized in the municipality’s main town or even outsourced to a neighboring municipality. This meant that much time was spent on public transport, as no refugees initially could afford a car, nor did they have a driving license that was legally accepted. In addition to the language training, most refugees had to do work training, which could be anywhere in the municipality or outside the municipality. This could often mean even longer commuting hours, as the public transport rarely connected the smaller towns. For those who also had to deliver children to kindergartens or schools, which were not always found in the same small town of residency, even more time would be spent on public transport. In the Norwegian case, among the families that were placed in the smallest of the three municipalities, the parents had to commute by bus for 2 h to get to their place of work training.

The very busy everyday lives, with long commuting hours, were described by many as frustrating and difficult, spurring feelings of disorientation. The refugees could find help to figure out timetables and commuter cards from their municipal caseworker, but, as they only saw them every couple of months, those refugees who had found local people to help them or had other refugees around to ask for advice felt much less frustrated and alone. Some had joined locals who drove them to the doctor or to see other refugees in other towns. Most refugees could not get their driving license accepted and had to take a new test and theory course, which was expensive and difficult in a new language. So, the lack of a car limited and made difficult their everyday movement. It was frustrating, but it also made them feel different. As several said, “*All the locals have at least one car, as living in a rural area is dependent on having a car … so having no car makes you really stand out*.” It took a few years before any of the refugees could start driving. Those that finally got a car found rural life much easier and less stressful, as commuting was a big part of their daily life for the first years.

Acquiring a driving license, and particularly driving lessons, represented an obstacle for some of the informants, particularly women living in a small village in the north of Norway. A couple of women reported that the only driving instructor in the village where they lived did not want to give lessons to people who were not Norwegian. The women had therefore to go to another municipality in order to have driving lessons; going there took more than 1 h by bus, each way. Taking driving lessons was too time-consuming for one of the women who had younger children, and she was therefore in a way “stopped”, as Simonsen, (2012) also describes, in her effort to get a driving certificate.

### Experiencing the Physical and Windy Rural Area

Several refugees describe the first months in their new places as very dark, cold and windy. The hours of darkness and the harshness of the weather differ enormously between the north of Norway and Denmark; nevertheless, the stories told about the encounters with nature and particularly the weather were surprisingly similar among the refugees settled in these different areas. Most refugees compared the wind and darkness to the lit-up city life and warm weather in their home countries.

Many refugees connected the darkness and the wind to feelings of loneliness and insecurity. The weather was also described as “uncomfortable” by several, who reported how they always walked quickly when they were outside to avoid the wind and rain from “*cutting through me*”, “*hitting me*” or “*slicing my skin*”. A young father in a Danish town said: “*I feel like not going outside, as the darkness seems intimidating and scary. …When it then also rains and the wind pushes me around, I feel like giving up*.” His wife suffered from depression on arrival, and he very much related it to the darkness and the feeling of loneliness and insecurity emanating from the dark winter.

One of the Syrian women who was settled with her family in a very small town in Norway stated that coming to this place in the late autumn was a huge challenge; the Sun had almost disappeared from the horizon and the autumn storms came one after another. The family had three children attending different levels at the local school, and the school was located 1 km from the house where they lived. As there was no bus and they could not get their driving license accepted, the children had to walk, often forcing themselves through severe weather along a dark road in order to get to the school. The mother said that they were all shocked and afraid of the windy weather. Walking from home to school in the early mornings, in wind and darkness, was very frightening. Thus, the distances are felt even more strongly when they have to be walked in harsh weather.

In these kinds of cases, with frustrated and frightened children, both the parents and children experienced moments of disorientation, where “The world was almost turned upside down” (Ahmed, 2006; Simonsen, 2012). As Ingold observes: “Indeed a strong wind can so overwhelm the senses as virtually to drown our perception of contact with the ground” (Ingold, 2010:131). The mother with the frightened children said that she often doubted whether they had done the right thing in bringing their children to such an area and perhaps it would have been better to stay in Syria, despite the war.

The severe weather had made an impression on everybody, and almost all respondents brought it up in interviews, even though it was not a question, to start with. There was a tendency for groups from a more rural background to also adapt more easily to the weather. A Somali mother of eight children in the Norwegian case said: “*The weather is rough, but we just put jackets on*.” Through bodily experiences, she changes or adapts her practice, as referred to by Kinkaid, (2020), while, for others, especially the former city-dwellers, the wind and darkness were brought up as key arguments for why they had plans to leave for a bigger town or city. Their comments may illuminate their experience of spatial disorientation and bodily alienation that mark their everyday experience, when their “normative meanings and practices they know from home cannot be used” (Kinkaid, 2020:180). If they give up on these schemes or practices, they might end up being seen as part of the mass of uneducated refugees who, for example, are portrayed in the media.

### Familiarization With Small Town Life

On the question of how the refugees used and perceived their new town and neighborhood, many of them felt that a good neighborhood was one that had lively street life and meeting places to go to, which the women especially missed in their new towns. Common in both cases is that the refugees find their new places of residence very quiet, and they compare them to their lively city backgrounds.

In the Norwegian case, those who came from larger cities reported that life in rural areas in Norway was too different from the daily life they were used to; in particular they found that the local people moved so slowly. The whole atmosphere in their new town felt quiet and slow, which was in opposition to how they experienced the pace of their own new everyday life, which was very busy, with commuting, language classes and job training. In the Danish case, respondents also said that they felt out of place when they went outside. There were very few people in the streets, and therefore they felt stared at when they moved around in bigger groups. In the Norwegian case, a woman reported that she stopped wearing her hijab in order to be more anonymous and not create attention as she moved around the town. The hijab marked her in a way that she wanted to avoid: an expression of embodied difference and otherness. In the Danish case, respondents placed in social housing expressed greater satisfaction with their homes and immediate neighborhoods, as buildings looked like the buildings other people lived in, and, secondly, because there were common spaces around the houses where they felt “allowed” to sit and meet neighbors and other refugees. Several respondents in both cases said that they found it difficult to “read” the towns, and they were unsure where you were “allowed” to sit down, as there were no obvious meeting places.

The busy, urban environment most refugees came from represented a strong contrast to the life they experience in their Danish and Norwegian small towns, in relation to weather, pace of life and meeting places but also in relation to the number and formality of social relations. In both the Danish and Norwegian cases, most of the informants had developed relations with other refugees (if there were any) in the towns: relations that they valued very highly. They described that meeting up with others in the same situation as themselves finally made them feel connected and not standing out. They spoke the same language, which made them able to open up and find common understanding. The relations to other refugees very much added to their feeling of being home and gave “peace”. These relations were initially more familiar and less formal than those they found in the small towns. Many of our informants mentioned that they felt unfamiliar with the new way of socializing by meeting in sports clubs and associations. They had noticed that the club houses and sports halls were the main meeting places and started to understand that it was here that social life played out, as they could not find many people in the streets. The women especially compared this way of socializing with the very different and informal way people met in the streets at home, where the children played in the streets after school. You could just go outside and immediately you would meet neighbors and friends you could talk with. On the contrary, in their new places of residence, they were expected to join associations and the children to do sports after school or to go to people’s homes for play dates. Several said that they were too shy to join an association and start doing sport, and many said they were also too busy in their new daily life and were thus in a way “stopped”, or at least their possibilities of social contacts were reduced, by their schemes and habits.

### Social Life in Between the Formal and Informal

The initial habits and schemes of the respondents in inhabiting their neighborhood and interacting with people in a less formal way made them feel unfamiliar, different and out of place in their new towns, at first. They felt unfamiliar with joining associations but felt more comfortable joining various activities and events to welcome refugees, set up by local volunteers. In the early days, it was events with food, music and dance. Later on, various collections of clothes and furniture for the refugees took place then came language and homework cafes and later recurring social cafes. “*Here in Denmark, we drink coffee with the Danes every third week at the social cafe*”, an older family father said with a smile, as this more organized way of socializing felt in contrast to the more informal way he met with the other refugees in the town. Even so, the social activities set up by local volunteers were in most cases popular among the refugees, in both the Norwegian and Danish cases. Most of the refugees spoke warmly about the people they met there. Those not taking part were mainly the younger single men.

Local activities seem important for the re-orientation of refugees after feelings of frustration and insecurity, dealing with busy everyday lives and dark winters. They represent important arenas for contact and interaction between the local population and the newcomers, as well as an entry point into the local social life that is more accessible for them than taking part in the associational life. However, most often, in both cases, the local volunteers were retired people; they were therefore somewhat older than the majority of the refugees. Our interviews with single men revealed that this group especially stopped taking part in the social activities after some time, mainly because of the age difference. They said that they felt shy and sometimes uncomfortable when having to speak to the older local people. “*I am sure they are nice, but I feel shy because what should I say to an elderly Danish lady?*” The young men may well feel inferior, uneasy and unfamiliar in chatting with an old woman who lives a completely different life from theirs. It is easier if the volunteers talk about practical issues or can help with insecurities about jobs and education rather than just coffee drinking and chitchat. Other reasons refugees have for stopping taking part in activities included whether they felt they could get help with some of the challenges they faced. A young father in the Danish case found that there was too much “hygge” (Danish word for a homely and cheerful atmosphere) at the social café, so he did not feel like bringing up subjects like his wife’s depression and his efforts to raise money to start up his own shop. In both the Danish and Norwegian cases, it was obvious that the social cafes whose volunteers had a network and a background in social work or teaching ran very professional activities for refugees. Here, they helped the refugees with legal aid and contact with the municipality, as well as advice on job finding, education and family reunification, whereas, in towns with mainly retired volunteers, the café activities were more for “hygge” and drinking coffee.

In both the Danish and Norwegian cases, younger locals also took part in the first activities for refugees, such as communal dinners and gatherings. In many cases, they dropped out after a while, mainly because of busy everyday lives but also because they found the responsibility too great to take on, as the refugees struggled with many and also complex matters in their daily life. As one Danish local volunteer said, “*It is much easier to mobilize younger people for collections and casual social activities than having to feel responsible for whether a refugee gets reunited with his family or to help out with jobs.*”

### Experiencing the Rural Context (Differently)

In the Danish case, it was very much the housing situation that refugees experienced as challenging. They were mainly accommodated in different “left-over” housing stock, such as kindergartens or nursing homes no longer in use. In the Norwegian case, the availability also of family oriented housing seemed greater, as people were to a larger degree settled in ordinary houses, but many found that the houses were in a bad condition. In the Danish case, being placed in left-over housing gave several of the refugees a feeling of their situation being temporary and therefore insecure. Much of the available housing, like left-over nursing homes, was best suited to the settlement of single people, as it was made up of single rooms. If a refugee was then reunited with his family, the municipality was required to find them a bigger home, which often meant that they had to leave the small town because there was no more suitably sized cheap rented housing available. A local Danish volunteer said: “*Our old nursing home has become a ghetto for those who cannot afford to move on, and it is a pity. Families find it easier here, but we have no housing for them*.” Therefore, most families had to leave, even those that had built social relations and had grown to like living in the town.

Most of the single people would like to move to bigger towns or cities for education and jobs; however, most in this group had no funds or contacts in the cities that could help them out with a place to live and a job. In the Danish case, it was especially the single men who would like to leave for better job opportunities. In Norway, there were also several women with children who were eager to move, in order to have the possibility of higher education. The refugees are free to move, even before the introductory years are over, but then they must pay deposits and moving expenses themselves and also find a place that they can afford, making it almost impossible for them.

In both cases, it was difficult and expensive to move, but many reported they had to, in order to find jobs and especially education. So, the distinction raised earlier between former city-dwellers and rural residents is also an important marker for those who feel they have to leave. Refugees with higher aspirations and education found it difficult to become familiar with and feel at home in the small town; even the weather was felt to be more of an issue for this group. Several of the refugees with higher education, particularly from Syria, could not get their academic degrees accepted for jobs they were qualified for and would have to undertake further education. However, as there were no higher educational possibilities where they were settled and commuting to places that had higher education was very expensive and too far away, moving away would be the only way to “make a life” in their new country. In the Norwegian case, most had to travel very long distances for education, even to the capital of Norway, Oslo, to take university courses that were relevant to their educational background. The only solution was to move and settle down in a new place again. Thus, the structural limitations-the distances, the housing and the lack of education possibilities-were very present in the refugees’ everyday life, often making them feel frustrated and that they were only in these areas on a temporary basis.

## Discussion

In the introduction to this paper, we referred to the debate on whether refugees should be settled in rural areas, and there is no simple answer. The empirical findings in our studies show that there are several “moments of disorientation” when settled in the Nordic rural environment. According to Simonsen, (2012), migration could be described as a process of both disorientation and reorientation, as the bodies both “moved away” and “arrived”. On arrival in the Nordic rural areas, there are many situations which the refugees do not know how to navigate, and many of their daily matters are in the hands of others. The adult refugees have very busy everyday lives, filled with language classes and job training and commuting outside the local area for most of the day. When they are finally back in the new rural place of residence, they find few people in the streets and are unsure where they are “allowed” to meet. The dark winter days, filled with rain and snow, make them want to stay indoors. These different moments in their new everyday lives evoke various feelings and sensory experiences like confusion, frustration, stress and insecurity.

The “physical” rural area, in the sense of limited outdoor public spaces, can make the towns difficult to “read” and inhabit for the refugees. In the literature on refugees in rural areas, there are some studies describing the lack of physical meeting places and how this hampers the possibility to establish local social relations between refuges and locals. The lack of physical meeting places limits the performance of everyday practices like shopping and taking children to school, which are important for developing a feeling of belonging and familiarity (Brekke, 2015; Feist et al., 2015; Nørregaard, 2018). In our study, the physical surroundings are compared by the respondents to the lively and informal city life at home. The small-town life and layout is unfamiliar, and the respondents can feel “exposed” when they move around in bigger groups. However, the physical rural environment is more than this, as Ingold (2010) says. The weather is present in the lives of the newly settled refugees. The “air”, in the form of wind, darkness and rain, evokes experiences and feelings that can be unsettling and challenging, just as other everyday meetings with commuter cards and housing matters can be. The Nordic weather, especially in Northern Norway, is a very challenging feature in the lives of the refugees. The weather and darkness can stir up senses and feelings of loneliness and insecurity. The body certainly walks, breathes, feels and knows in the weather world. When it comes to the phenomenology of practices, the way the rural areas are experienced from within shows surprisingly many similarities, when it comes to the experiences of transport and communication, surroundings, social relations and even the weather.

It is more across different groups of refugees that the weather and the rural setting are experienced differently. The former city-dwellers, with higher levels of education and job aspirations, find the small-town residency a temporary place on their way toward a better life with more possibilities in the city, and they are more resistant to changing their spatial practice and habits. The structural conditions of a rural setting very much shape the everyday lives of refugees in the first years, when they do not have a car or the financial capacity to buy their own house.

Living in small town presents many of the same challenges for refugees as for Danes and Norwegians. Many local young people leave rural areas for education and more possibilities but structural factors might be even more difficult to overcome when living on refugee benefits and when one is unfamiliar with the “vibrant” but somehow hidden and formal small town civic life. As referred to in the introduction, the limiting structural factors, like lack of jobs, housing and social services, are very much brought forward as difficult in the lives of refugees in rural areas (Wren, 2003; McAreavey and Argent, 2018; Woods, 2018). The rural setting is difficult to adapt to but some refugees might adapt better if they do not have aspirations for city living and higher education.

Our study shows that, when people and their “bodies” cannot use acquired schemes and habits, they can feel “exposed”, constrained and out of place, which are all examples of Kinkaid, (2020) “*differences formed through lived practice*”. Kinkaid, (2020) used the term “contradictions of space” when people cannot practice space in a way that they are familiar with and where the relation between the subject and the milieu fails to cohere. Such experiences can result in both spatial and social disorientation, “*As the normative meanings and practices they know from home cannot be used*” (Kinkaid, 2020:180). In our material, we find few respondents actually having been “stopped” in their everyday spaces, as Simonsen, (2012) sees in nightlife, but most respondents definitely have their possibilities reduced, feel strange, exposed, looked at or out of place. Their “visibility” and the process of “othering” is more subtle and set in their comparing themselves to what is done around them and sometimes modifying their spatial practice accordingly. The woman that stopped wearing her hijab is the most explicit example of adjusting regarding visibility. The hijab was an expression of embodied difference, as it marked her in a way that she wanted to avoid. Most refugees notice that they live in different houses from those of the locals around them, have no car, travel by public transport, unlike local adults, etc.: all conditions that they cannot do much about. To get a driving license is a way out, but it is expensive and time-consuming, especially if the local driving instructor “stops” people by not wanting to drive with foreigners. We do not know the reason for the driving instructor not driving with foreigners. It might be the language, or it might be racism, but no matter what it exacerbates the problem of long commuting hours for the respondents and “stops” those already involved in many daily trips to bring children back and forth. The respondents also feel unsure about their spatial practice of inhabiting their new town of residence, as mentioned above. How should they move around and engage socially with the locals? If they live in social housing with public meeting places and can meet at events for refugees rather than at sports, they feel more at ease.

The best solutions for the many situations and feelings of not knowing what to do and how to find out seems to be social relations with other refugees and the local community. The experiences and feelings Simonsen pointed to a feeling of meaning and belonging, feeling of home, familiarity and orientation and of making the space habitable–can be spurred by socializing, especially with other refugees. Socializing with other refugees is highly valued and evokes feelings of familiarity. Feelings of both shyness and unfamiliarity characterize the social meetings between the refugees and the local community at the beginning. The way people meet in sports clubs and associations feels strange and formal, whereas the activities set up for refugees by local people seem more accessible. Here, they can get advice and ask for help without feeling bad, but it is not in all towns that the social atmosphere encourages them to get help with more challenging matters.

Recent literature on the local communities’ role in migrant integration is two-sided. If they are not active and welcoming, it is a factor that could make immigrants leave (Skaptadóttir and Wojtynska, 2008; Johansen, 2018; Woods, 2018), and if they are too active, they can end up holding on to refugees that might have better opportunities elsewhere (McAreavey and Argent, 2018; Woods, 2018). Studies from Nordic rural areas point to the fact that interaction with the host community is key to a feeling of attachment and belonging, but establishing this interaction can be challenging (Herslund, 2021). Studies of unaccompanied young refugees in Sweden and Norway have shown that limited interaction with local youth leads to a lack of commitment among young refugees (Brekke, 2015; Wernesjö, 2015). The same can be found in studies on labor migrants in fishing and farm villages, where limited interaction with the local population makes this group feel less attached to their new residence (Skaptadóttir and Wojtynska, 2008; Aure et al., 2018). Scholars have also pointed out that relations with and proximity to other migrants are important in the feeling of belonging (Larsen, 2011). They can act as mediators between newly arrived refugees and the host society and ameliorate the drawbacks of rural residency, like limited social services and sparse economic opportunities (Larsen, 2011).

In our cases, the local communities have all been active, but often it is in drinking coffee or practical matters, whereas it can be harder to mobilize people to help out with more difficult matters. In addition, the local community cannot solve all problems. They can help ameliorate some of the obstacles of being settled in a rural town, by helping out with transport, but they cannot change the limited amount of cheap rented accommodation characterizing rural towns, nor can they change the darkness and wind that are so very different from where the refugees come from.

The question is, of course, whether one can talk about “Nordic” rural areas as one entity. It is widely known that rural areas can be very different, and rural areas in a small country like Denmark and a large and much more northern country like Northern Norway definitely present very different conditions. Especially, we have seen how distances in Northern Norway pose many difficulties for refugees in their daily life. This is to a lesser extent the case in Denmark, but even here there are challenges, when one does not have a car like everyone else. What surprised us was how the weather was experienced as harsh and very intimidating in both countries by refugees. Coming to small places in northern Europe, from urban areas and places in the Middle East with heat and Sun, represents great changes. It is not surprising that the refugees struggle, living in such different weather conditions. In Northern Norway, there are a couple of months where the Sun is above the horizon. The polar night and the winter can be hard, even for people who have lived there all their lives.

## Conclusion

This paper is based on empirical data gathered in two different projects, studying refugees settled in rural areas in the north of Norway and in Denmark. Inspired by the phenomenology of practices (Simonsen, 2012), which situates practical, embodied consciousness in the world, we asked the questions: When arriving in Nordic rural areas, how is space (the new town of residence) experienced, what experiences result in feelings of belonging and familiarity, and what experiences spur feelings of disorientation?

Despite great differences in geography and climate across the two cases, the informants in our studies encounter many of the same challenges to do with the rural environment: the long distances and limited public transport, few meeting places, different behavioral norms, unfamiliar weather conditions and more formal social interactions, etc. Being settled in Nordic rural areas has produced moments of contradictions, where the relation between the subject and the milieu has failed to cohere: situations of not knowing how to navigate. Being in a situation where many daily matters were in the hands of others also created disorientation and demanded reorientation. Another common factor in the lives of the families and young people settled in both Norway and Denmark was the busyness of their everyday lives. The necessity to commute outside the local area most of the day, in order to participate in the introductory program, having work practice, take driving lessons, etc., was rather time-consuming. Many of the informants also expressed feelings of disorientation, since there were so few people out in the streets.

This paper has pointed out that refugees settled in rural Norway and Denmark can experience many similar challenges. Nevertheless, there is an important difference among those with refugee status in both the north of Norway and the south of Denmark. The former city-dwellers, with higher levels of education and job aspirations, find the small-town residency difficult in both cases, whereas refugees with rural backgrounds more easily feel at home. Still, such differences seemed to be played down, in encounters with both local people and others with a refugee background.

Taking part in the introductory program with others in the same situation, speaking a “foreign” language, living in particular houses and being unfamiliar with local culture and habits created important boundaries and experiences of familiarity among the refugees. At the beginning of the settlement phase, experiences of insecurity, shyness and unfamiliarity and not knowing how to practice space appropriately made it difficult to getting to know local inhabitants. It was difficult to find and approach locals, in both the small town informal local arenas and the more formal associational life and sports clubs. Most of the encounters between newcomers and local people took place at activities set up especially for refugees. Many of our informants expressed great gratitude for the locals engaging in such activities, as these were more accessible ways into the local community. However, these activities also needed to address the many challenges the refugees experienced in their daily life. If they are only centered around “hygge” and drinking coffee, they lose out and might not be prioritized by the refugees, as they have busy everyday lives. Encounters with the Nordic rural areas created moments of disorientation and the experiences and feelings that Simonsen, (2012) points to; feelings of meaning and belonging are mainly spurred by socializing, especially with other refugees in the same situation but also by taking part in local voluntary activities, helping out with everyday life challenges.

## Data Availability

The article include original contributions. Further inquiries can be directed to the corresponding author.
